# An approach to capturing the cultural contexts of health behaviours

**DOI:** 10.1007/s00103-025-04105-6

**Published:** 2025-08-11

**Authors:** Veerle Snijders, Katrine Bach Habersaat, Robert Böhm, Edward Fischer, Felicity Thomas, Julie Leask, Martha Scherzer, Alona Mazhnaia, Nils Fietje

**Affiliations:** 1 Behavioural and Cultural Insights Unit, WHO Regional Office for Europe, UN City, Marmorvej 51, 2100 København, Denmark; 2 Department of Occupational, Economic, and Social Psychology, Faculty of Psychology, University of Vienna, Vienna, Austria; 3 Department of Banking and Finance, Faculty of Business and Management, University of Innsbruck, Innsbruck, Austria; 4 Department of Anthropology, Vanderbilt University, Nashville, TN USA; 5 Department of Health and Community Sciences, University of Exeter, Exeter, UK; 6 School of Public Health, Faculty of Medicine and Health, University of Sydney, Sydney, Australia

**Keywords:** Social determinants, Arts and health, Participatory research, Public health, Community, Soziale Determinanten, Kunst und Gesundheit, Partizipative Forschung, Öffentliche Gesundheit, Gemeinschaft

## Abstract

This paper explores the relationship between cultural contexts and health behaviours, emphasising the need for a comprehensive understanding of cultural influences and acknowledging the limitations of current approaches to health behaviours in unpacking cultural dimensions of health behaviours. We argue that health behaviours, which are integral to achieving good health and health equity, are deeply intertwined with the cultural frameworks within which they occur. In response to an increasing recognition among health authorities of the importance of health behaviours and their cultural dimensions, the paper discusses (1) the role of culture in shaping health behaviours, (2) the intersectionality of cultural identities, and (3) the systemic factors that contribute to health inequities. Using the World Health Organization Tailoring Health Programmes approach as a framework, we propose an approach to health programmes and policies that is behaviourally informed and culturally grounded. By advocating for a participatory, community-engaged approach and emphasising the importance of disaggregated data, the paper aims to inform future public health strategies that are more equitable, responsive, and effective in promoting health behaviours across diverse populations.

## Abstract (de)

In diesem Beitrag wird die Beziehung zwischen kulturellen Kontexten und Gesundheitsverhalten untersucht, wobei die Notwendigkeit eines umfassenden Verständnisses kultureller Einflüsse hervorgehoben wird. Zudem werden die Grenzen aufgezeigt, die derzeitige Ansätze zur Erforschung des Gesundheitsverhaltens haben, wenn es darum geht, dessen kulturelle Dimensionen zu entschlüsseln. Gesundheitsverhalten ist für gute, chancengleiche Gesundheit unerlässlich, und wir argumentieren, dass es eng mit den kulturellen Rahmenbedingungen verwoben ist, in denen Gesundheit auftritt. Als Reaktion auf die zunehmende Anerkennung der Bedeutung von Gesundheitsverhalten erörtert der Artikel: (1) die Rolle der Kultur bei der Beeinflussung des Gesundheitsverhaltens, (2) die Überschneidung kultureller Identitäten und (3) die systemischen Faktoren, die zu gesundheitlichen Ungleichheiten beitragen. Auf der Grundlage des WHO-Konzepts für maßgeschneiderte Gesundheitsprogramme (Tailoring Health Programmes, THP) schlagen wir einen Ansatz für gesundheitsbezogene Programme und politische Maßnahmen vor, die sowohl verhaltensorientiert als auch kulturell verankert sind. Wir plädieren für einen partizipativen, gemeinschaftsorientierten Ansatz und betonen die Bedeutung von nach Subgruppen aufgeschlüsselten Daten. Dadurch soll der Artikel Informationen für künftige Public-Health-Strategien liefern, die gerechter, reaktionsfähiger und effektiver bei der Förderung des Gesundheitsverhaltens in unterschiedlichen Bevölkerungsgruppen sind.

## Introduction

Many health challenges facing the world today are considered preventable and treatable. Tackling these requires more than new technologies and medical discoveries such as insights into the behaviours and cultural contexts that produce them. The ways behaviour influences health, and how cultural contexts affect behaviours, are often complex and intertwined. Classic behavioural science, rooted in psychology and behavioural economics, focuses on individual determinants of behaviour such as perceptions, attitudes, subjective norms, and intentions. Such models may place an undue responsibility on individuals because they underemphasise the influence of physical and social factors as well as cultural contexts. Today, widely used health behavioural models and theories include social and cultural dimensions; however, they are often limited in their ability to unpack and operationalise these dimensions. Integrating tools and methods from the medical humanities and social sciences can help address this challenge. This is not a simple exercise, as the cultural dimensions can be more dynamic and fluid and therefore more complex to capture and address. Health authorities are increasingly looking for opportunities and tools that can help address the behavioural dimensions of public health challenges in a way that captures diverse cultural influences.

## Behaviour is integral to good health

Over the last two decades, governments worldwide have increasingly applied behavioural insights to a variety of policy areas, including health. [1, 2]. This trend reflects a growing recognition of the complex, holistic determinants of health in which behaviour plays a crucial role—from illness prevention and health promotion to the treatment and management of disease. The COVID-19 pandemic further highlighted the importance of behaviours like handwashing, mask-wearing, and vaccine uptake. Even “upstream” actions, such as new legislation or hospital strategies, are driven by individual and group motivations, professional environments, resources, and structures.

Health behaviours are intrinsically linked with health equity. Noncommunicable diseases are strongly associated with the conditions in which people grow up, live, and work, including poverty, unemployment, substandard housing, and poor physical environments [3]. Limited health literacy is associated with increased levels of risk behaviours and low adherence to recommended health behaviours [4]. Understanding and integrating cultural contexts into health interventions can help reduce disparities by tailoring services to disadvantaged populations [3].

Recognizing this, the World Health Organization (WHO) Regional Office for Europe prioritized “behavioural and cultural insights” (BCI) for health. In September 2022, the 53 regional member states adopted a resolution and action framework for BCI, committing to more effective approaches for addressing health behaviours [5]. This was followed by a global resolution to use behavioural sciences for better health [6]. These resolutions promote a people-centred approach that seeks to understand the individual and contextual factors affecting health-related behaviours and use these insights to develop more effective evidence-based policies, services, and communications, combined with a rigorous evaluation of such interventions [7].

## Culture shapes health behaviours

Health and illness are often thought to be internal to the individual. However, broader systems, including market structures, political values, physical environments, and cultural contexts, interact with individuals and communities, shaping their health behaviours. In 2014, the Lancet Commission on Culture and Health concluded that “the systematic neglect of culture in health and healthcare is the single biggest barrier to advancement of the highest attainable standard of health worldwide” [8]. Cultural contexts affect health and well-being in profound, and often hidden, ways. This goes beyond cross-cultural communication and cultural competency. Social hierarchies result in excess morbidity and mortality for marginalized peoples [9]. Cultured gender and sexual norms can create vulnerabilities and increase the transmission of disease [10]. Yet, just as culture can contribute to harm, it also offers opportunities to promote health and well-being.

In 2015, the WHO Regional Office for Europe established its first expert advisory group on the cultural contexts of health and well-being, aligning with the 2030 Agenda for Sustainable Development. This agenda emphasises that achieving the sustainable development goals (SDGs) requires addressing health determinants such as social status, environment, and support networks with cultural sensitivity.

The understanding that culture significantly impacts health outcomes has gained traction in recent years. In 2015, the WHO Regional Office for Europe established its first expert advisory group on the cultural contexts of health and well-being. This aligned with the broader aims of the 2030 Agenda for Sustainable Development, adopted by all UN members that same year, which promotes a more holistic understanding of health determinants, including income and social status, education, gender, physical environment, social support networks, genetics and health services [11, 12]. Achieving the SDGs requires careful attention to these cultural contexts, as one-size-fits-all solutions often fail to address the nuanced realities of culturally diverse populations [13].

In fact, it has been argued that “studying the ways in which systems interact to create health or illness is the leading edge of a revolution of understanding in medicine” [14].

Despite its importance, culture is frequently oversimplified in behavioural frameworks [15]. For instance, the Behaviour Change Wheel and its COM‑B model (Capability—Opportunity—Motivation-Behaviour) reduce culture to a “social opportunity” that “dictates the way we think about things” [16]. Similarly, the commonly used theory of planned behaviour [17] and the EAST (Easy, Attractive, Social, and Timely) framework [18] consider culture only implicitly through constructs such as social factors and subjective norms. It is only recently that contributions to behavioural science have begun to recognise the role of culture—not only in influencing behaviour but also in shaping how researchers design studies and interpret findings [19]. This growing recognition marks a step towards more contextually grounded, culturally informed insights into human behaviour.

## What is culture in relation to health behaviours?

The complexity in defining culture may be one reason why it is underutilised in health programming. The United Nations Education, Scientific and Cultural Organization (UNESCO) defines culture as “the set of distinctive spiritual, material, intellectual and emotional features of society or a social group … (which) encompasses, in addition to art and literature, lifestyles, ways of living together, value systems, traditions and beliefs” [20]. Culture is understood here as the shared and dynamic values and assumptions that frame our sense of reality and give us a moral direction. Culture goes beyond ethnic, religious, racial, or national association [11], permeating everything we do. We are all products of our culture: It influences our values, beliefs, social norms, and practices. As such, we all have culture and live in a cultural context, creating and reproducing ways of being, including our institutions and research fields. Because cultural contexts are so central to all population groups and their health experiences, understanding these contexts is critical if we are to inform (and even change) health behaviours.

### Communities and subcultures.

While culture is often equated with national identity (e.g., French culture or Azerbaijani culture), it is important to recognize the cultural heterogeneity within countries and societies. Culture is not exclusive; individuals participate in a variety of cultures simultaneously. Within countries we find different cultural patterns across ethnic groups, migrant and refugee populations, religious traditions, urban and rural communities, and myriad other cultural groups. What might seem like a good policy for a majority population may not work for other subcultures. For example, water, sanitation, and hygiene policies that work well for urban, white Slovenian communities may not have the same impact on Roma population groups who hold different cultural experiences and living realities [21].

Other subcultures, such as men who have sex with men (MSM), may be particularly proactive in efforts to prevent disease. Since the mid-1990s, many MSM have engaged in serosorting as one method of HIV prevention, i.e., choosing sexual partners of the same HIV status to reduce the risk of transmission [22, 23]. While serosorting is most effective where HIV testing is frequent, status is shared honestly, and condoms are not consistently used, this approach developed within the MSM community itself, highlighting the importance of community engagement in finding culturally appropriate solutions.

### Intersectionality of culture.

It is important to recognise that a person may identify with multiple cultures, such as belonging to a migrant group and having a set of religious beliefs while also being part of the LGBTIQ+ (lesbian, gay, bisexual, transgender, intersex, queer/questioning) community [24]. Each of these cultures will influence the person’s behaviour, and the norms of each culture may interact and collide [25].

The PEN‑3 cultural model considers such intersectionality and can be useful for exploring three cultural domains and their impact on health behaviour [26]. The first domain considers the cultural identity of the individual and how that may interact with the identity of their family, social networks, and community. The second domain is relationships and expectations, emphasising social norms and social support systems and how these influence the perception of the health behaviour. The third considers cultural empowerment and how cultural practices can promote, be neutral, or hinder health behaviours [26]. The PEN‑3 model is mostly used in health communication and education, such as COVID-19 communication and HIV/AIDS communication [27, 28], providing a useful tool for identifying complex contextual factors and the potential for the positive impact of culture. While it would benefit from more rigorous evaluations [29], it may supplement widely used behavioural models. For example, it can help in exploring and better understanding the nuances of the social opportunity factors when using the COM‑B model [16].

### Individual and contextual culture.

It can be helpful to distinguish between an individual’s cultural background, which influences their values, beliefs, and preferences, and the cultural context they live in, which includes their physical and social environments. For health behaviours, it is beneficial to place additional emphasis on the cultural context over the individual culture. For example, regarding childhood obesity, it is likely that individual cultural beliefs play a role, such as body image, food preferences, and exercise habits [30]. However, focusing on individual choice or a lack of self-control can be ineffective or even harmful [31]. Instead, a more productive approach considers the cultural and physical context that people inhabit. How does the local food environment influence dietary behaviour? How do commercial systems reinforce food habits by tapping into cultural beliefs? How do built environments support or hinder physical activity? Successful interventions address these contextual factors, using strategies such as marketing constraints, taxes, and nutritional labelling [30]. Another example of creating more conducive social and cultural environments for health-related behaviours concerns waste practices. Traditionally, policymakers have predominately relied on information campaigns to encourage people to reduce, reuse, and recycle their household waste. A recent evidence synthesis highlights the important role cultural contexts play in these waste practices. It shows how strategies such as community-led clean-up efforts and tailored communication that resonates with lived experience can help reframe waste from something that has lost all meaning to something that can be meaningful again [32].

### Threats to cultural identity.

Systematic threats to one’s culture—whether through colonisation, war, or migration—can have detrimental effects on people’s health. In Greenland, the legacy of colonisation and cultural disruption has been linked to increased rates of alcohol consumption and suicide [33, 34]. Conversely, efforts to rebuild and strengthen cultural identity have shown protective effects, particularly among First Nations youth in Greenland, Canada, and the United States, where cultural reconnection has been associated with reduced trauma and lower suicide rates [35, 36]. Overall, with First Nations health, a request is often voiced to respect sovereignty, start with a blank page, and meet community needs to truly co-design and support indigenous health workers in their roles. Acknowledging and incorporating cultural identity and environmental context into health interventions is therefore essential for promoting positive health behaviours in these populations [34]. Similarly, for migrant and refugee communities, the impact of a loss or destruction of culture should also be considered a critical determinant of health behaviour. Understanding this can help identify more effective, culturally sensitive, and restorative public health strategies [11].

### Organisational culture.

While we often consider culture to be something that other people have, we recognise that scientists and medical professionals are also influenced by their cultural contexts. Organisational culture refers to the unique set of ideas and behaviours shared among members of an organisation [37]. By creating a collective mindset—or one that gives dominance to a powerful subset—organisational culture shapes behaviours in public health settings. Strong organisational cultures can negatively influence outcomes if poor practices become normalised through “cultural entrapment” [38]. For instance, whether an organisation considers health a strictly biomedical concept or utilises a more holistic approach by integrating social influences will affect recommendations and prescriptions. Complex organisational structures, such as hospitals and health care institutions, rely on the successful interaction of multiple individuals and processes, based on shared norms of behaviour and expectations—key components of organisational culture [39, 40]. Consequently, organisational culture impacts patient safety and hospital performance [41], physician decision-making [42], compliance with infection prevention procedures [43, 44], the uptake of new guidelines [45], the sharing and learning of mistakes [45], and the health and well-being of both patients and staff [46, 47]. Where a “culture of blame” pervades an institution, health care workers may avoid reporting adverse events for fear of punishment by management or legal authorities [48, 49].

### Cultural competence of health systems.

Institutional culture is related to cultural competency, i.e., the ability of a health system to accommodate people with diverse cultural backgrounds [50]. Key elements of influencing culturally competent care include the quality of the leadership, the diversity of the workforce, and the nature and quality of interactions and communication between health care workers and patients. When these elements are lacking, care can become culturally insensitive, exacerbating health disparities.

Cultural competency can be strengthened through a variety of strategies, such as increasing minority representation through targeted recruitment, providing education and training on cultural differences and sensitivities, and offering interpreter services or even cultural mediators [50]. Cultural mediators can act as a bridge between the cultures to translate between the health care system and people and are often used for migrant or refugee populations who may have different health care expectations. These mediators translate both aspects, not just languages but also by helping health workers and patients understand each other’s world views, perceptions, values, and norms, and they can help direct people to the right services to improve access or convey health behaviours, recommendations, and advice between health care workers and patients [51]. The concept of “cultural safety,” a term first coined by Māori nurses in Aotearoa, New Zealand, takes this one step further by critically reflecting on one’s own culture and existing power structures and how those may influence clinical interactions and behaviours [52]. These approaches aim to provide better care through culturally sensitive models.

## Challenges of applying culture to health behaviours

Although combining behavioural and cultural approaches can provide better insights into the determinants of health, their misapplication carries important risks—particularly when culture is misrepresented and misinterpreted.

### Researcher bias.

Researchers may inadvertently fall into stereotypes and assumptions that result in missing more important systemic factors influencing health behaviours, such as geographic and economic constraints. An example of this is highlighted by a tailoring health study aiming at improving vaccination coverage among children of the Charedi Orthodox Jewish community in London, who had lower immunisation rates than their peers [53]. Assumptions about cultural or religious antivaccination sentiments were ill founded. Instead, the research identified that the critical barriers were more pragmatic, such as travel time to the clinic, the small waiting areas that did not have sufficient space for larger families, and the limited opening hours. A campaign focusing only on cultural barriers would likely have failed [53].

An additional bias may be perceiving culture as a barrier rather than a strength. Culture can be supportive of health behaviours, and interventions that align with cultural norms can help people to utilise cultural resources in support of the behaviour [25, 27, 54]. Participatory research with Scottish Muslim women identified how faith, in combination with education, can be used as an asset to promote awareness and acceptability of cancer screening [55]. It has also been found that the Black church can create a culturally and spiritually sensitive support system for the mental health and related behaviours of Black males in the United States [56].

### Rigid data structures.

Aggregated data can hide a variety of inequities within the health system [57]. Capturing disaggregated data can show how behaviours and interventions impact differently across subgroups and cultural identifiers. By being aware of inequities and differences in health and health behaviours, we can more effectively understand and address them. Although many of these differences may have complex determinants that require long-term and systemic changes, even small interventions can lead to improvements. In a replication of a successful social norm letter intervention to decrease the overprescribing of antibiotics in the United Kingdom [19], researchers in New Zealand had a particular challenge: Māori and Pacific populations had lower antibiotic prescribing rates despite having poorer health and a greater risk of infections, i.e., there was likely a problem of both overprescribing for some population groups and underprescribing for others. A more complex version of the letter was tested, which included prescribing rates for both majority and minority populations. The intervention was successful in decreasing the prescribing of antibiotics overall, while increasing prescribing for these at-risk groups [58].

## How to incorporate cultural perspectives to health

A culturally contextual approach challenges the limitations of conventional “social determinants” frameworks, which often imply linear, mechanistic relations between specific determinant and health outcomes. In reality, cultural contexts are dynamic, fluid, and open-ended—meaning that the same determinant can lead to different outcomes depending on the setting. This complexity highlights the need for health programmes and interventions to be tailored to specific cultural and contextual realities of the populations they aim to serve.

To illustrate more specifically when and how a cultural dimension can be integrated into health behaviour work, we present the WHO Tailoring Health Programmes (THP) approach as an example. The THP approach was developed as a guide to understanding and addressing health behaviours [31]. Structured across four phases, the THP approach supports users in identifying and understanding both individual and contextual factors that shape health-related behaviours. Two core principles are emphasised throughout: “tailoring” health policies to specific needs of communities, rather than relying on one-size-fits-all solutions, and a “contextual” perspective that situates behaviour within broader physical, political, social, and cultural contexts, thus moving away from a narrow focus on individual responsibility [31]. To guide the analysis and interpretation of behaviour, the approach uses a modified version of the COM‑B model, which considers how capability, physical and social opportunity, and motivation act as facilitators or barriers of health behaviours [7, 16]. Within this model, the cultural context is captured as a social opportunity. Below, we describe how this dimension can better be unpacked, drawing on the reflections above.

### Phase 1: Situation analysis.

Situation analysis involves reviewing existing knowledge and data and defining the target group and behaviours. To ensure proper attention to the cultural context of the health behaviour, the social, religious, cultural, and equity dimensions of the target populations should be considered. Stakeholders who represent a wide range of interest groups should be approached. In addition, it is helpful to consider academic perspective from outside traditional public health fields, including research from the health-related humanities (such as medical history, cultural studies, and, of course, anthropology and other social sciences).

### Phase 2: Research.

During this phase, research is planned and conducted to address knowledge gaps, with a particular focus on the barriers and drivers to the target behaviour. The cultural dynamics can be explored by incorporating the voices of affected communities in the research, such as through in-depth interviews and focus groups. Observational studies, such as rapid ethnographies in which researchers observe people in their natural settings, can be particularly useful for gaining insights into topics where self-reported answers might be less reliable. When the goal is to explore the perspectives and interests of research participants within their own familiar cultural contexts, arts-based data collection may be the preferred approach. This method avoids exposing participants to research methodologies that may be alienating to them. Arts-based data collection can involve various art forms, such as visual arts, audiovisual media, performative arts, creative writing, and storytelling [59]. One example is photovoice, which allows participants to document and reflect on their experiences through photographs rather than (or in addition to) spoken or written words [59].

### Phase 3: Intervention design.

In this phase, research insights are translated into an intervention. To ensure an appropriate cultural dimension in this phase, interventions should be tailored to the community through their active engagement. This may be achieved through co-design workshops, feedback sessions, patient participation groups, or knowledge dialogues. Such participatory processes promote the incorporation of cultural contexts and benefit the ownership, acceptance, and sustainability of the policy or intervention. Where written or verbal feedback is not sufficient, exercises to map the people or patient journeys and body maps can be applied. The latter involves a “life-sized” human body image on which someone can draw, paint, or otherwise visually represent a person’s life, body, health, behaviours, and world they live in. It is also worth noting that cultural engagement itself can be a health intervention [60]. For example, using verbatim theatre to express impacts of workplace mistreatment of students and junior health professionals can improve workplace culture [61].

### Phase 4: Implementation and evaluation.

Finally, in the last phase, the intervention is implemented, and its long-term impact on the target behaviour is monitored and evaluated. This includes evaluating whether the intervention has different impacts on different people, whether these differences are grounded in cultural dimensions, and whether applying a cultural lens can help address potential shortcomings. In fact, much like the intervention design, a thoroughly co-created evaluation can help reduce researcher bias and increase stakeholder buy-in. Even with robust evaluations [61], there is a risk that quantitative studies can miss the positive or negative impacts, or the lack of impact, in subgroups. At the same time, while an intervention may successfully reach its behavioural targets, there may be unintended stigmatisation or negative impacts on the well-being, trust, or sense of social cohesion of those affected—or the intervention may not reach the original targets but may have unexpected positive effects. Qualitative evaluation through, for example, in-depth interviews can shine a further light on the impacts in key subpopulations and broaden the scope of evaluation from the specific behavioural outcome to essential broader aspects.

## Conclusion

**Fig. 1:**
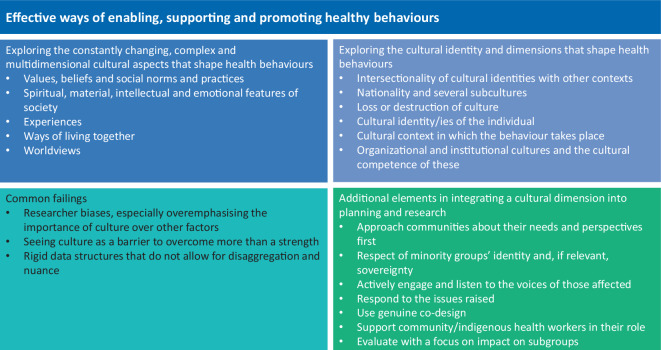
Matrix for considering the cultural contexts of health behaviours

Commonly used models and frameworks for understanding health behaviours often do not adequately incorporate cultural dimensions. This limitation poses a significant challenge, hindering our ability to fully explore the barriers and drivers of health behaviours and to design truly effective interventions. As outlined in this paper, culture encompasses not only values, beliefs, norms, and practices but also the spiritual, material, intellectual, and emotional characteristics of a society. These cultural elements shape our perceptions, lived experiences, and ways of relating to others, ultimately influencing health behaviours in profound and multifaceted ways (Fig. 1). Given the complexity and dynamic nature of culture, more sophisticated models are needed that can account for its multidimensional effects on health behaviours. These models should inform how cultural insights can be translated into practical strategies for enabling, supporting, and promoting healthier behaviours.

In this paper, we emphasised different types of cultural contexts and identified several cultural pitfalls that must be avoided. To effectively support and promote healthy behaviours, we must take all these aspects into account and engage directly with affected communities. This includes listening to their lived experiences and evaluating interventions with an emphasis on equity and impact across subgroups. However, this is only the beginning of a journey towards a more fundamental understanding of the cultural context of health behaviours and ways to capture these. More work is needed to develop cultural models that can complement existing behavioural frameworks, to test these through research and implementation, and to integrate them into curriculum. Only by integrating these nuanced cultural perspectives can we design more responsive, equitable, and effective public health interventions.

## Declarations

For this article no studies with human participants or animals were performed by any of the authors. All studies mentioned were in accordance with the ethical standards indicated in each case.
